# Comparison of longitudinal variance components and regression-based approaches for linkage detection on chromosome 17 for systolic blood pressure

**DOI:** 10.1186/1471-2156-4-S1-S17

**Published:** 2003-12-31

**Authors:** Mariza de Andrade, Curtis Olswold

**Affiliations:** 1 Division of Biostatistics, Department of Health Sciences Research, Mayo Clinic, 200 First Street, SW, Rochester, Minnesota, USA

## Abstract

We compare two methods to detect genetic linkage by using serial observations of systolic blood pressure in pedigree data from the Framingham Heart Study focusing on chromosome 17. The first method is a variance components (VC) approach that incorporates longitudinal pedigree data, and the second method is a regression-based approach that summarizes all longitudinal measures in one single measure. No evidence of linkage was found either using the VC longitudinal approach or the regression-based approach, except when all time points were used from Cohorts 1 and 2 and only subjects aged 25 and 75 years were included.

## Background

Modeling becomes more complex when observations are recorded over time. Several authors have developed and reviewed statistical methods for longitudinal cohort studies [1]. For genetic analysis, Province and Rao [2,3] used path analysis to estimate the genetic and environmental effects in families in the presence of temporal trends or a time effect, but did not include variance components (VC) to measure effects from specific genes. Models using structural equations have also been developed and widely applied in the field of behavioral genetics to twin studies [4-6], but these models, primarily directed to the estimation of polygenic and environmental effects, are difficult to use for studies of large families or extended pedigrees. Huggins and colleagues [7] applied cubic spline methods to analyze longitudinal data in twins, but their method considered only additive polygenic effects. Recently, de Andrade et al. [8] proposed an extension of the VC approach to incorporate longitudinal family data. Another approach to analyze longitudinal family data is to use regression methods ignoring the family structure, and then use the residuals as the quantitative traits. Several authors [9,10] have proposed different variations of this approach. The regression-based approach summarizes all longitudinal measures in one single measure and uses this summarized measure that is the regression residuals, for linkage detection, i.e., each individual has one single value that represents his/her longitudinal measures of blood pressure. On the other hand, the longitudinal VC approach does not summarize the longitudinal measures and uses them all, i.e., each individual has a vector of measures that represents his/her measure of blood pressure levels at each time point. We focus our analyses primarily on chromosome 17 because it was the chromosome where Levy et al. [9] showed strong evidence of linkage for systolic blood pressure. Therefore, the purpose of this paper is to compare these two approaches, the longitudinal VC approach proposed by de Andrade et al. [8] and the residuals approach proposed by Levy et al. [9], using the Framingham Heart Study data set.

## Methods

### Longitudinal VCs

The longitudinal variance components (LVC) approach is an extension of the VC approach proposed by Amos [11]. For longitudinal familial data, let **Y_i _= (Y_i1_,...,Y_iT_)' **be a vector of T time point trait values for k_i _members of the i^th ^family, where **Y_it_' = (Y_i1t_, ..., Y_ijt_,..., Y_ikit_)' **for t = 1,..., T. Let **E(Y_i_) = μ + X_i _β **and **V_i _= A****G_i _+ B****Π_i _+ C****I_i_**, where  defines the direct product of two matrices; **G_i _**is a k_i _× k_i _matrix of coefficient of relationship between pairs of relatives; **Π_i _**is a k_i _× k_i _matrix of IBD values for the i^th ^family; **I_i _**is a k_i _× k_i _identity matrix; and **A**, **B**, and **C **are, respectively, polygenic, major gene, and random environment variance-covariance matrices each of dimension T × T. These matrices are represented by **A **= (σ_a.tt'_), σ_a.tt _= σ^2^_a.t_, **B **= (σ_g.tt'_), σ_g.tt _= σ^2^_g.t_, **C **= (τ_g.tt'_), τ_g.tt' _= τ^2^_a.t_, with their typical elements in the parentheses. We assume **Y_i _**follows a multivariate normal distribution. More details about this method can be found in de Andrade et al. [8].

To test for genetic linkage, we construct a likelihood ratio test. Under the null hypothesis, the major gene parameters, the σ_g.tt _for all t and t', are restricted to be equal to **0**. The distribution of the longitudinal test is a mixture of χ^2 ^values [12]. For example, for two time points linkage analysis of an additive genetic effect, the distribution of the bivariate test that the major-gene covariance components are zero is a mixture of 1/4 χ^2^_0_, 1/2 χ^2^_1_, and 1/4 χ^2^_3_.

The longitudinal feature was incorporated in the software ACT [13] within the module multic, which was used to run the analyses. The longitudinal multipoint linkage analysis was performed only in the concordant five time points from Cohorts 1 and 2. The trait of interest was systolic blood pressure (SBP), and the covariates were age, gender, and body mass index (BMI). Individuals with missing values were eliminated from the analysis.

### Residuals approach

We used the two-stage procedure described in Levy et al. [9]. This procedure first calculates the within-subject mean BP, and second, uses the sample-wide regressions adjusted for age and BMI, yielding a residual for each subject. Then, these residuals are the traits used in the quantitative linkage analysis. In our analyses, we consider two cases: 1) all subjects in both cohorts regardless of age and 2) only subjects between 25 and 75 years. Each of these analyses was then stratified by time points: 1) using all 21 time points from Cohort 1 and all five time points from Cohort 2 and 2) using only the concordant five time points from Cohorts 1 and 2. After the residuals were obtained, multipoint quantitative linkage analyses were performed for these four different scenarios using SOLAR [14]. We also performed a multipoint quantitative linkage analysis using the average SBP over all measurements for each subject for additional comparison. LOD score values were calculated by dividing the likelihood ratio statistic by 4.6 (2/log e).

## Results

**Figure 1:**
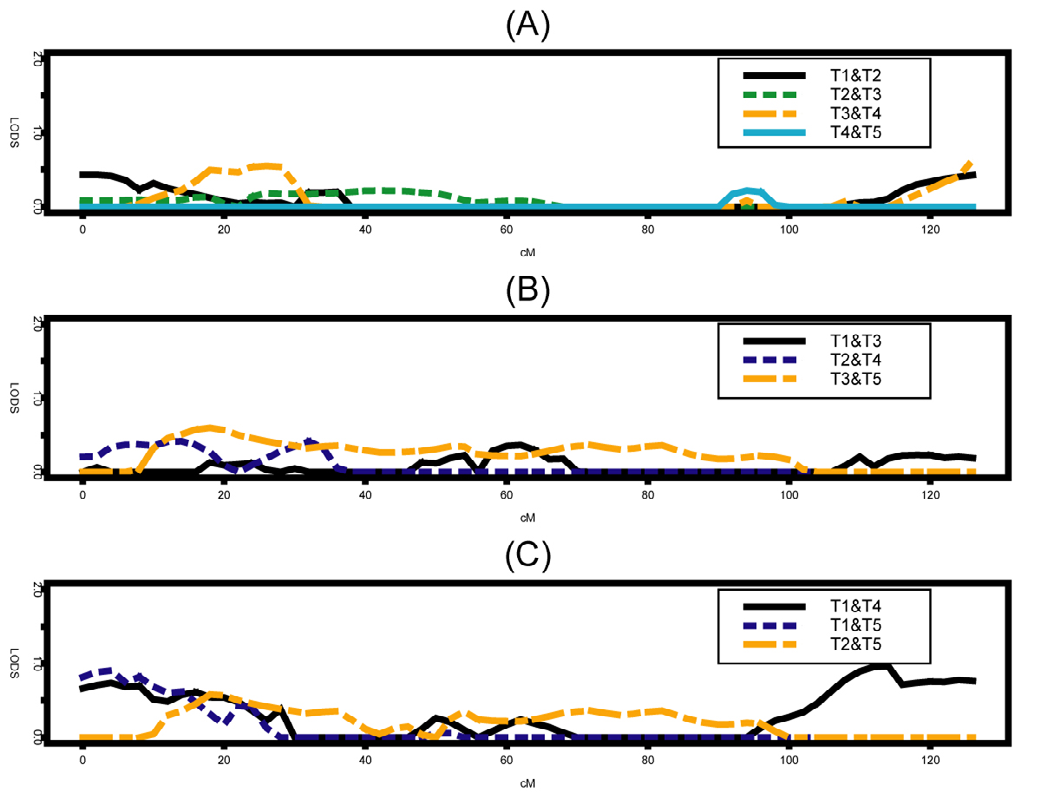
Three multipoint LOD scores plots using longitudinal variance components approach on chromosome 17 for pair-wise time points: at 5 years apart (A), at 10 years apart (B), and at least 15 years apart (C).

**Figure 2:**
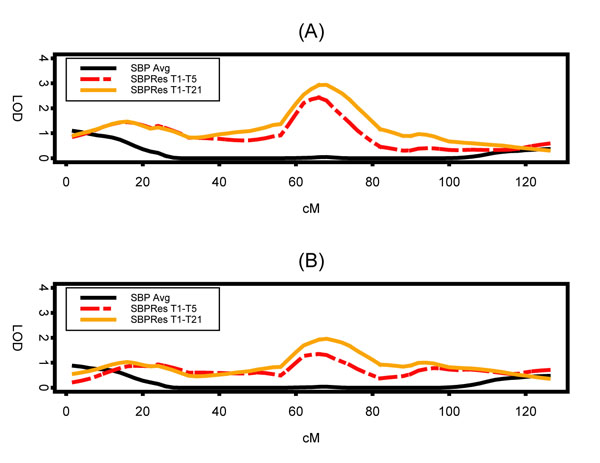
Two multipoint LOD scores plots using the residuals approach on chromosome 17 for three different analyses: using all 21 time points from Cohort 1 and all 5 time points from Cohort 2, only the concordant five time points from Cohorts 1 and 2, and the average SBP over all measurements for each subject: with age restriction from 25 to 75 (A), and using all ages (B).

Figure 1 shows the summary of the multipoint linkage analysis using the longitudinal VC approach for all possible pair-wise time points. Figure 1A,1B,1C, depicts the multipoint LOD values for pair-wise time points at 5 years apart, at 10 years, apart and at 15 years apart, respectively. No evidence of linkage was found when using the longitudinal family data structure. The maximum LOD was 0.96 in the surrounding region of the 114-cM position for time points 1 and 4 (Figure 1C). Figure 2 shows the multipoint quantitative linkage analysis using the residuals, i.e., the summarized measures calculated using the regression-based approach. The only evidence for linkage (LOD = 3) occurred when all time points (21 from Cohort 1 and 5 from Cohort 2) and only subjects with ages between 25 and 75 were used.

## Discussion

Although evidence of a gene influencing blood pressure was reported on chromosome 17 using the Framingham Heart Study [9], the findings (LOD = 4.7, position = 67 cM) could not be replicated in our analyses. Only when SBP from all time points in Cohorts 1 and 2 were used and the subjects' age were restricted to be between 25 and 75 years was a comparable finding reached (LOD = 3.0, position = 68 cM). Slager and Iturria [10] also could not replicate Levy et al.'s findings when using two other approaches of phenotype definitions. We also expected that the longitudinal VC approach would perform much better because it takes into account a temporal trend affecting the genetic variability of the SBP. However, the maximum LOD found using this procedure was around 0.96 in the surrounding region of the 114 cM position for time points 1 and 4 (Figure 1C). There was no consistency among the pair-wise time points multipoint LOD values.

The reason why the results of the two approaches differ could be several. It could be due to missing values, since individuals with missing values were eliminated from the analysis in the longitudinal VC but not from the regression-based approach. The amount of missing SBP values among pair-wise time points is around 50%, where time point 5 has more missing values than the other time points. Another explanation could be the adjustment that was used for treatment effect in SBP in Levy et al. [9], i.e., the inferred values could be larger than expected, thus inflating the LOD values [15]. In our longitudinal VC analysis and in our regression-based analysis we did not adjust for treatment effect. We assume that if the treatment works it lowers the SBP. Therefore, the impact of not correcting for treatment makes the linkage results more conservative, which is preferable to false-positive results. Finally, the departure of normality could also inflate the LOD values, i.e., increase the false-positive rate [16].

In summary, the longitudinal VC and the regression approach showed similar results when all subjects were used regardless of their age, i.e., no evidence for linkage was found on chromosome 17. However, the evidence for linkage was only observed when all SBP time points were analyzed from both cohorts and when subjects' age were restricted to be between 25 and 75. Therefore, one possible conclusion is that the linkage results depend heavily on the phenotype. By using the residuals as a summarized measure of the longitudinal SPB, the time point variability and missing mechanism are not taken into account and could therefore lead to spurious linkage results.
